# Spontaneous Rupture of a Parietal Arachnoid Cyst Causing an Intracystic Hemorrhage and a Subacute Subdural Hematoma

**DOI:** 10.7759/cureus.67280

**Published:** 2024-08-20

**Authors:** Alejandro E Cedeño-Morán, Orlando De Jesus

**Affiliations:** 1 Neurosurgery, University of Puerto Rico, Medical Sciences Campus, San Juan, PRI

**Keywords:** spontaneous, subacute, subdural hematoma, hemorrhage, intracystic, arachnoid cyst

## Abstract

Arachnoid cysts are extra-axial cerebrospinal fluid collections located in the arachnoid space that usually do not communicate with the ventricular system. They are commonly found in the middle cranial fossa around the Sylvian fissure. Most of them are asymptomatic, but subdural collections or intracystic hemorrhages can complicate their natural course. Cases of intracystic hemorrhage and subdural hematoma, especially in the absence of traumatic events, have been scarcely reported. Arachnoid cysts outside the middle cranial fossa are rarely associated with an intracystic hemorrhage. We present the case of a 10-year-old boy with a known right parietal brain arachnoid cyst who complained of progressive headaches for three days. There was no history of trauma. The head CT scan showed a 2-cm isodense subacute subdural hematoma, causing a mass effect on the underlying brain. It was associated with a right parietal arachnoid cyst containing intracystic subacute blood. The symptoms were relieved after burr-hole surgical drainage of the intracystic hemorrhage and associated subacute subdural hematoma.

## Introduction

Arachnoid cysts (ACs) are extra-axial cerebrospinal fluid (CSF) collections located in the arachnoid space that usually do not communicate with the ventricular system. They are believed to represent embryological malformations resulting in the compression of underlying brain tissue [1-3]. They account for 1% of all intracranial space-occupying lesions, occurring most commonly in the middle cranial fossa [1,3-7]. ACs frequently produce scalloping and thinning of the overlying bone [2,4,8,9]. Approximately 60%-80% of ACs are discovered before age 15, showing a male predominance [5]. Most ACs are asymptomatic and remain stable in size, often found incidentally through radiological studies. The increased use of MRI and CT scans has led to a rise in the diagnosis of these asymptomatic cases [1,3,4,8,10].

Symptomatic ACs can produce increased intracranial pressure, presenting signs or symptoms such as headaches, nausea, vomiting, and seizures [1,3-5,10]. Symptoms may occasionally be caused after a subdural hygroma or a subdural hematoma is formed when the AC ruptures [1-3,5,7,11,12]. Rarely does an intracystic hemorrhage occur and produce symptoms [1,2,5,11]. Subdural hemorrhages can occur spontaneously or after minor trauma; however, spontaneous intracystic hemorrhage is an uncommon phenomenon [1,5].

## Case presentation

**Figure 1:**
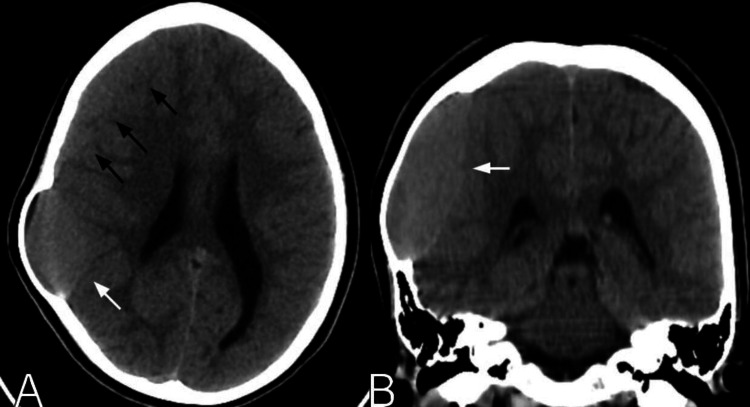
Preoperative head CT scan axial (A) and coronal (B) views showing a right isodense subacute subdural hematoma (black arrows) causing a mass effect on the underlying brain, associated with a parietal arachnoid cyst containing intracystic subacute blood (white arrows).

**Figure 2:**
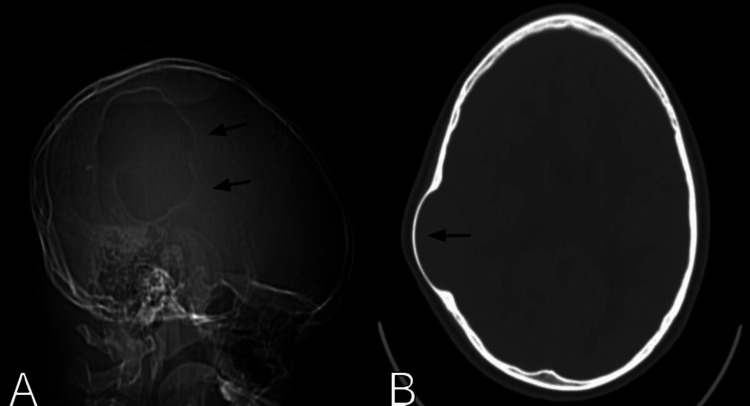
Head CT scan skull scout view (A) and bone window axial view (B) showing the classic bone thinning and scalloping (black arrows).

A 10-year-old male with a known right parietal brain AC diagnosed five years before is presented. The AC has been observed over the years, as the patient did not have any intracranial symptoms related to it. Three days before the admission, the patient complained of a frontal headache that initially improved with an oral analgesic. The following day, the patient's headaches became severe, which worsened upon lying flat and were associated with one episode of nausea and vomiting that did not resolve with oral medication. This episode prompted the mother to take him to the local hospital. A head CT was performed, which showed a 2 cm isodense panhemispheric subacute subdural hematoma associated with a right parietal AC containing subacute blood (Figure 1). The hematoma caused a mass effect on the underlying brain, resulting in a 7-mm right-to-left midline shift. The CT scout and bone window views showed the classic bone thinning and scalloping (Figure 2). The patient was transferred to our institution for neurosurgical evaluation and management.

**Figure 3:**
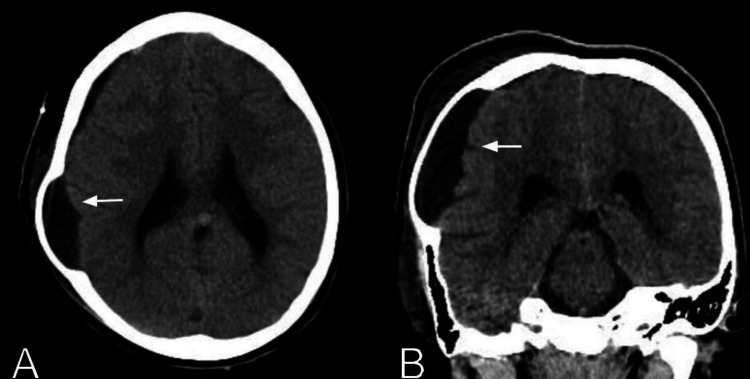
Postoperative head CT scan axial (A) and coronal (B) views showing complete drainage of subdural hematoma and intracystic hemorrhage, demonstrating the cerebrospinal fluid-filled parietal arachnoid cyst (white arrows).

Upon evaluation, the patient was awake and alert but complained of a severe headache. The patient and mother denied any recent trauma or forceful physical activities. Our assessment revealed a patient with a known right AC that ruptured and developed an intracystic hemorrhage and a right subacute subdural hematoma. He was taken to the operating room on an emergency basis. A right-side parietal burr-hole drainage was performed to drain the intracystic hemorrhage and subacute subdural hematoma. After the surgery, the symptoms resolved. The postoperative head CT scan showed complete drainage of both hemorrhages, revealing the CSF-filled AC (Figure 3). He was discharged home on the first postoperative day. At the six-month follow-up, he manifested no symptoms, and a head CT scan showed no recurrence of the bleeding with a stable AC size.

## Discussion

Most ACs are asymptomatic; however, those producing symptoms are typically associated with cyst enlargement, thought to occur by several mechanisms, including CSF being driven into the cyst through a ball-valve mechanism, an osmotic gradient between the cyst and adjacent CSF in the subarachnoid spaces, or secretion by the arachnoid cells within the cyst lumen [1,3,4,9,11,13]. Although ACs are frequently found near the Sylvian fissure, they can also be located in the parasellar region, cerebral convexity, interhemispheric fissure, quadrigeminal plate, cerebellopontine angle, vermian, and retroclival area [9,14]. Galassi et al. classified middle cranial fossa ACs into three basic types using a CT scan and metrizamide CT cisternography [15]. Type I is the mildest form, being small, spindle-shaped, or biconvex, and limited to the anterior aspect of the temporal fossa. Type II is the classic type, medium-sized and triangular or quadrangular-shaped, occupying the anterior and middle parts of the temporal fossa and extending superiorly along the Sylvian fissure. Type III is the most severe form, associated with a large oval or round cyst occupying almost entirely the temporal fossa.

Symptoms may also be attributed to the rupture of the AC. The rupture can produce subdural hygromas, subdural hematomas, or intracystic hemorrhages. For the correct diagnosis, an MRI is superior to a CT scan [11,13,14]. The MRI identifies the margins of the cyst and its effect on adjacent structures [14]. In our case, a brain MRI was not necessary for the diagnosis, as the CT scan clearly showed bone scalloping, subdural hematoma, and intracystic hemorrhage. Massimi et al. recently reviewed the literature on ruptured Sylvian ACs, identifying 446 cases, of which 28 developed an intracystic hemorrhage, for a 6.5% incidence [7]. In a study of chronic subdural hematomas and hygromas, Parsch et al. found that ACs of the middle cranial fossa were identified in 2.4% of the patients [8]. They estimated that for patients with ACs of the middle cranial fossa, the hemorrhage risk would not exceed 40/100,000 cases per year (0.04%) [8].

Sylvian ACs are much more likely to rupture than those at other locations [7]. ACs in other brain locations that produce hematomas are extremely rare [16]. AC rupture occurs more frequently in Galassi type II [3,7]. Male patients, usually under the age of 18 years, with middle cranial fossa ACs have a higher risk of rupture or bleeding [4,5,7]. In the study by Wu et al., the mean age of patients with ACs associated with subdural hematomas was 24 years, with 53% of the patients under the age of 20 [12]. The mechanism for intracystic hemorrhage and subdural hematoma is attributed to the rupture of intracystic or bridging vessels, particularly veins that may tear with minimal or trivial trauma [6,10,16]. Tearing small bridging vessels between the dura and outer membrane of the AC may produce subdural or intracystic hemorrhage [9,10,16]. These blood vessels surrounding the ACs have a fragile supporting stroma, which makes them prone to rupture and bleeding [6,9]. In cases where there is no history of head trauma, spontaneous tearing of the AC wall during sports events or forced physical exercises can occur, leading to the rupture of the cyst membrane or adjacent bridging veins, with leakage of CSF or blood into the subdural space [17,18].

Treatment approaches for AC rupture complications are still controversial [12]. A burr-hole drainage of the subdural hematoma is usually sufficient to relieve the symptoms as it permits drainage of the subdural collection and the intracystic hemorrhage. The burr hole should be placed over the AC in those cases showing an intracystic hemorrhage. If a membrane separates the AC from the subdural space, the membrane can be opened, allowing the drainage of the subdural hematoma and intracystic hemorrhage. However, this treatment does not eliminate the AC and carries the risk of rupture recurrence. An external drainage is frequently placed through the burr hole for a few postoperative days to clean the collection from any residual blood. External drainage may be optional in cases where the irrigation was clean at the end of the procedure.

Some authors suggest that cyst fenestration into the skull base cisterns is the most effective procedure for addressing the acute compression and recurrence risk [5,13]. Fenestration can be done endoscopically or using a craniotomy. In our opinion, cyst fenestration is not required in patients whose AC was asymptomatic before the development of the intracystic hemorrhage. Wu et al. published an extensive review of ACs associated with chronic subdural hematomas, reporting an 8.2% recurrence rate of the subdural hematoma with burr holes and a 1.5% recurrence rate with craniotomy [12]. Sayer et al. noted a change in the surgical approach for treating subdural hematomas associated with AC, with the significant use of burr holes in recent times instead of craniotomies [19]. Although still controversial, conservative management with active surveillance has been suggested for ruptured ACs with minimal symptoms since the collection can sometimes resolve spontaneously [8,12].

## Conclusions

Cases of intracystic hemorrhage and subdural hematoma, especially in the absence of traumatic events, have been scarcely reported. ACs located outside the middle cranial fossa rarely produce an intracystic hemorrhage. The combination of a parietal AC associated with intracystic hemorrhage and subacute subdural hematoma is uncommonly seen, especially if traumatic events are absent. Consensus on the treatment method for symptomatic cases has yet to be established in the literature. In the case presented, burr-hole drainage of the intracystic hemorrhage and the subacute subdural hematoma was adequate to relieve the acute symptoms. For asymptomatic patients in whom surgical fenestration was not done, active surveillance of the AC is recommended as it can re-rupture or grow. The prognosis for patients sustaining the rupture of an AC is favorable.
